# Dr. Frederic Skey Stanwell

**Published:** 1910-07-09

**Authors:** 


### Obituary.

We have to announce the death of Dr. Frederic Skey Stanwell, aged thirty-seven. He studied at Edinburgh University, took his M.B. and C.M. in 1895, and graduated M.D. ten years later. He had been house physician to the Edinburgh Royal Infirmary, and previously assistant medical officer to the East Riding Asylum, Beverley, and to the Northern Hospital, Winchmore Hill, and North East Hospital, Tottenham.

